# The Modulatory Effects of Anesthetics and Analgesics on Neurophysiological Monitoring and Underlying Mechanisms

**DOI:** 10.2174/011570159X349119250127104107

**Published:** 2025-02-18

**Authors:** Yu Leng, Yi Teng, Jin Liu, Xian Zou, Mengchan Ou, Tao Zhu, Peng Liang, Cheng Zhou

**Affiliations:** 1 Department of Anesthesiology, West China Hospital, Sichuan University, Chengdu, 610041, China;; 2 Research Center of Anesthesiology, National-Local Joint Engineering Research Centre of Translational Medicine of Anesthesiology, West China Hospital, Sichuan University, Chengdu, 610041, China;; 3 Day Surgery Center, West China Hospital, Sichuan University, Chengdu, 610041, China

**Keywords:** Intraoperative neurophysiological monitoring, somatosensory evoked potentials, electroencephalography, neurological function, anesthesia, analgesia

## Abstract

Intraoperative Neurophysiological Monitoring (IONM) is an indispensable surgical tool that offers invaluable insights into neurological function across a spectrum of anatomical areas. By comprehensively assessing the integrity of the brain, brainstem, spinal cord, cranial nerves, and peripheral nerves, IONM plays a pivotal role in guiding surgical decision-making and optimizing patient outcomes, particularly in the context of high-risk procedures. Intraoperative drugs, especially anesthetics and/or analgesics, differentially modulate neurophysiological monitoring, which remarkably affects the application of neurophysiological monitoring under specific conditions and indicates the neurobiological mechanisms of anesthetics/analgesics. This review will describe various neurophysiological modalities utilized in intraoperative procedures, each employing a wide variety of physiological principles; summarize the modulatory effects of anesthetics/analgesics on these neurophysiological monitoring parameters; and elucidate their underlying mechanisms, with a particular emphasis on evoked potentials. Insights gleaned from this review can inform strategies of anesthesia management for surgeries that require IONM and guide future investigations on the mechanisms of anesthesia/analgesia.

## 1 INTRODUCTION

Prior to the first use of intraoperative neurophysiological testing [1], sensory and motor functions can only be assessed by clinical observation (*e.g*., the wake-up test). Recent developments in the field of neurological monitoring have made it possible to evaluate neurological functions more effectively and objectively [2-4]. The importance of preventing an unsuspected and/or unpleasant neurological deficit after surgery has been emphasized. Intraoperative Neurophysiological Monitoring (IONM) is commonly used in the operating room to improve surgical decision-making and patient outcomes. When applied correctly, IONM can be used to measure the functional integrity of neural pathways, thus enabling early detection of compromised neuronal structures and timely intervention before the injury becomes permanent.

Several neurophysiological modalities are currently available for monitoring various aspects of the central and peripheral nervous system, each offering a unique set of benefits, limitations, and sensitivity/specificity as diagnostic techniques [5]. Among the current IONMs, the most frequently used modalities are electroencephalography (EEG), somatosensory evoked potentials (SSEPs), motor evoked potentials (MEPs), spontaneous electromyography (sEMG), and triggered electromyography (tEMG). Compared with each modality alone, multimodal IONM is more sensitive and specific for predicting neurological injury [5-8]. Hence, IONM is an attractive option to ascertain, in real-time, the well-being of the nervous system during high-risk interventions. However, intraoperative neurophysiological monitoring is susceptible to a variety of factors, of which the most important are the anesthetics/analgesics used perioperatively. In recent years, an increasing number of studies have investigated the mechanisms of action of general anesthetic drugs, exploring their pharmacological effects from molecular targets and cellular processes to neural circuit level [9-14]. However, how these mechanisms of action of anesthetics and/or analgesics contribute to their ability to modulate neurophysiological monitoring remains to be elucidated.

This review summarizes the effects of anesthetics/analgesics on neurophysiological monitoring parameters and briefly describes the possible underlying mechanisms involved. This review will specifically stress the use of evoked potentials to analyze the effects of anesthetics/analgesics on neuronal pathways in order to provide insights for anesthesia management during procedures that involve IONM and for future research on the mechanisms of anesthesia/analgesia.

## 2 PHARMACOLOGICAL EFFECTS OF ANESTHETICS/ANALGESICS ON INTRAOPERATIVE NEUROPHYSIOLOGICAL MONITORING (IONM)

Anesthetics can exert direct effects on the nervous system and have a significant influence on neuromonitoring parameters, thus affecting the interpretation of the results of IONM. Therefore, an optimal anesthesia regimen is necessary to ascertain whether neurological function is impaired during neurophysiological monitoring. The true status of IONM can only be determined accurately and in a timely manner after considering the effects of anesthetics/analgesics.

### 2.1 Electroencephalography (EEG)

Electroencephalography (EEG) is widely used to investigate the spontaneous electrical activity of the cerebral cortex and does not reflect subcortical activity [15]. The electrical signal is amplified, filtered, and displayed during EEG recording to provide an accurate representation of cortical electrical activity [15]. Multiple scalp electrodes allow monitoring of a large surface area of the cerebral cortex, with each electrode offering a continuous view of a spherical region approximately 2 cm to 3 cm in diameter. While scalp EEG reflects the spontaneous electrical activity of the cerebral cortex, it does not capture activity from deeper structures. Typically, eight, 12, or 16 scalp electrodes may be utilized [2, 16].

Coordinated action potentials, also known as spikes, are essential mechanisms for information exchange within the brain and central nervous system. Neurons transmit and receive these spikes, generating extracellular electrical potentials primarily consisting of postsynaptic potentials and neuronal membrane hyperpolarization. These extracellular potentials, often termed local field potentials (LFPs), play a crucial role in coordinating and modulating communication within and among neural circuits, frequently exhibiting oscillatory patterns. LFPs generated in the cortex can be measured at the scalp as electroencephalogram signals [17]. Different anesthetics interact with various molecular sites and/or neural circuits, inducing diverse states of altered arousal and distinct EEG signatures. EEG-based monitoring is utilized to assess anesthesia depth during procedures, with the bispectral index (BIS) being one of the simplest and most commonly employed methods [18]. Previous studies have demonstrated the ability of the BIS to improve intermediate outcomes during the operative period, such as reduced hypnotic drug administration, shorter extubation time, decreased postoperative nausea, and faster recovery room discharge [18].

EEG patterns are affected by changes in the oxygen supply and neuronal perfusion. When Cerebral Blood Flow (CBF) drops below 25-30 ml/100 g/min, the EEG exhibits frontal fast frequency reduction [19]. A further reduction in perfusion to 18-25 ml/100 g/min leads to an increase in slow wave activity, beginning with theta waves and progressing to slower delta waves in the 12-18 ml/100 g/min range. EEG depression and electrocerebral silencing occur when the CBF falls below 10-12 ml/100 g/min and are often accompanied by disruption of the neuronal membrane [19, 20]. Based on these EEG changes associated with CBF levels, EEG has been proposed as a technique for monitoring the brain during cardiovascular surgery [15]. Although EEG can be used to assess the cerebral cortex for ischemia during intracranial surgery, surface EEG recording may not reliably detect subcortical ischemia [21]. Additionally, the induction of iatrogenic burst suppression during temporary arterial occlusion hinders EEG monitoring for detecting cerebral ischemia during critical phases of surgery. EEG interpretation can also be complicated by the use of anesthetic agents and coexisting hypothermia [15, 21].

## 3 MOLECULAR TARGETS AND EFFECTS OF ANESTHETICS AND ANALGESICS ON EEG

This review will only briefly introduce the effects of anesthetics on EEG patterns, and the details of the specific mechanisms involved can be found in the relevant literature that focuses on EEG under anesthesia [17, 13, 22-25].

### 3.1 Propofol

Propofol is the most widely used intravenous general anesthetic. The molecular mechanism of propofol has been well characterized. It exerts an inhibitory effect mainly by enhancing or directly activating postsynaptic GABA_A_ receptors and partly by enhancing glycine receptors [26-29] or inhibiting HCN channels [30]. Propofol may also directly modulate neuronal excitability by inhibiting persistent currents of Na_v_ and L-type high-voltage activated calcium channels [31], thus inhibiting presynaptic neurotransmitter release [32]. Overall inhibition is caused by GABAergic enhancement and/or interruption of glutamate transmission. Since GABAergic inhibitory interneurons are widely distributed in the cortex, thalamus, brainstem, and spinal cord, propofol induces unconsciousness by augmenting GABAergic inhibition in these brain regions [17]. For example, in the cortex, propofol decreases synaptic transmission by enhancing GABA-mediated inhibition of cortical pyramidal neurons. Moreover, propofol interrupts thalamocortical communications by enhancing GABAergic inhibition at the thalamic reticular nucleus (TRN), an important source of inhibitory regulation of thalamic output [17]. These inhibitory effects can contribute to the beta and alpha oscillations of the EEG patterns under propofol anesthesia [33]. In addition, propofol can enhance GABAergic inhibition from the preoptic area of the hypothalamus (POA) to other wake-promoting areas in the brain, which decreases excitatory inputs to the cortex and contributes to the slow and delta oscillations of EEG pattern [33, 34]. Like other anesthetics, a high dose of propofol can cause burst suppression in EEG signals [17], which is characterized by alternating periods of high-amplitude electrical activity (“bursts”) and nearly complete suppression of electrical activity (“suppression”). These phenomena may be associated with extracellular Ca^2+^ depletion and the ability of neurons to restore this concentration [35].

### 3.2 Dexmedetomidine

Dexmedetomidine is a selective α2 agonist and is often used as an adjunct in the operating room. Compared with patients who receive other narcotic hypnotics, patients who receive dexmedetomidine are characterized by easier arousal and little suppression of respiratory function. For the action mechanism, dexmedetomidine binds to presynaptic α2 receptors in locus coeruleus (LC) neurons and reduces the neuronal firing rate and norepinephrine release in a concentration-dependent manner [36]. The decreased norepinephrine input from the locus coeruleus affects many arousal-related nuclei, such as the basal forebrain [37]. In addition, the hyperpolarization of locus coeruleus neurons results in the loss of inhibitory inputs to the preoptic area of the hypothalamus (POA), which sends inhibitory projections to major arousal areas. This disinhibition effect on the POA may be essential for NREM sleep [38, 39]. It may further disrupt thalamocortical functional connectivity [40], thus leading EEG patterns to a combination of slow-delta oscillations with spindles under dexmedetomidine sedation, which resembles physiological sleep [41]. Low-dose dexmedetomidine infusion may induce a sedative state in which patients are easily aroused. Dexmedetomidine infusion resulted in a smaller amplitude of slow oscillations in EEG and fewer states of neuronal silence than did propofol infusion [42], which suggests a lower level of disruption in neuronal activity.

### 3.3 Benzodiazepines

Benzodiazepines produce sedative, hypnotic, anxiolytic, and myorelaxant effects. Recent studies have shown that benzodiazepines may also have analgesic and anti-depressive effects [43, 44]. Primarily, benzodiazepines modulate the opening of chloride channels on GABA_A_ receptors, which are activated by GABA neurotransmitters [45]. Hallucinations caused by ketamine can be treated with benzodiazepines, which is consistent with this GABA-mediated mechanism [46]. Midazolam, for instance, has been observed to augment delta band amplitude in EEG while diminishing occipital alpha amplitude, reminiscent of the effects of propofol anesthesia [47, 48].

### 3.4 Ketamine

Ketamine induces both hypnotic and analgesic effects and primarily operates by blocking postsynaptic NMDA receptors in the brain and spinal cord [49]. These actions result in the preferential inhibition of NMDA receptors on GABAergic inhibitory interneurons, thereby disinhibiting downstream excitatory neurons. Such aberrant excitatory effects may contribute to the hallucinations, dissociative states, and euphoria induced by ketamine [50]. The activation of cortical pyramidal neurons then causes EEG signals to display fast oscillations in the high-beta and low-gamma range under ketamine [17]. Neural field modeling indicates that the effects of ketamine on EEG rhythmicity in the theta, alpha, and beta bands are largely attributable to its activity at HCN channels rather than its primary effects on NMDA receptors [51]. Connectivity analyses of electrophysiological data revealed that ketamine preserves or enhances feedforward and thalamocortical connectivity while disrupting feedback and corticocortical connectivity. This disruption may reflect a reduction in sensory feedback, consistent with a decrease in beta spectral power in the EEG pattern [52].

### 3.5 Etomidate

Etomidate, which acts as a modulator and mimetic of GABA, exhibits high selectivity for GABA_A_ receptors, with minimal or negligible effects on other receptor types, such as neuronal nicotinic, 5-HT3, glycine, or kainate receptors [53]. By targeting synaptic GABA_A_ receptors, etomidate can potentiate or directly activate inhibitory postsynaptic currents, thereby enhancing inhibitory neurotransmission [54]. Additionally, at clinical concentrations, etomidate can activate GABA_A_ receptors extrasynaptically, leading to an increase in tonic inhibitory “leak” current and a subsequent reduction in neuronal excitability [55]. For EEG patterns, etomidate-induced anesthesia is associated with heightened theta-wave oscillation and robust coherence in theta waves, likely attributable to the potentiation of GABA_A_ receptor β3 subunits [56, 57]; however, etomidate also depresses theta oscillations *via* β2-subunit-containing receptors. These alterations in theta frequency and power may contribute to the development of amnesia through anesthetic actions on various molecular targets [58], most notably hippocampal theta rhythms. The theta oscillations may indicate a functional disconnection between the hippocampus and cerebral cortex [57].

### 3.6 Opioids

Opioids serve as common adjuncts during surgical procedures and postoperative pain management. They predominantly bind to endogenous opioid receptors, including μ, δ, κ, and nociceptive receptors, all of which are G protein-coupled receptors [59]. Activation of these receptors can potentially reduce exocytotic release by blocking presynaptic voltage-dependent Ca^2+^ channels and hyperpolarizing the neuron membrane through the opening of G-protein-gated inward rectifying potassium channels (GIRKs) [60, 61]. For EEG, both fentanyl and alfentanil elicit similar EEG changes characterized by progressive frequency slowing and amplitude increases [62]. This suggests that these opioids have impacts on brain activity comparable to those of general anesthetics.

### 3.7 Volatile Anesthetics

Since the first ether anesthesia was introduced, understanding the relationship between their actions at the molecular level and the behavioral effects of volatile anesthetics has become the main goal of mechanism studies of anesthesia. Volatile anesthetics are widely used in clinical practice because of their faster induction and/or recovery process. The mechanisms of action of volatile anesthetics remain to be fully explored, and several studies have shed light on the possible molecular targets of these agents. Like other intravenous general anesthetics, volatile anesthetics can inhibit neuronal activity *via* GABA_A_ agonism [63]. However, GABA_A_ receptor activation does not fully explain the effects of volatile anesthetics. For example, they can antagonize NMDA receptors and/or modulate two-pore domain K^+^ channels, further inhibiting excitatory synaptic transmission [64-68].

Similar to propofol, the volatile anesthetic sevoflurane induces coherent frontal alpha oscillations and slow oscillations in humans during the anesthesia-induced unconscious state, suggesting potential shared molecular targets and neural mechanisms of cortical information processing between sevoflurane and propofol [69]. The EEG patterns under sevoflurane anesthesia transit from robust alpha oscillations and slow-delta oscillations to prominent theta oscillations bridging the delta and alpha bands as the concentration increases from subminimum alveolar concentration (sub-MAC) to MAC [17]. Conversely, theta oscillations tend to diminish as the concentration decreases, which may suggest a non-GABAergic mechanism and is indicative of profound thalamic deafferentation that acts through low-threshold T-type calcium channels in thalamic neurons [69].

## 4 EVOKED POTENTIALS

Evoked potentials (EPs) are electrical potentials generated in response to stimulation of the nervous system, whether through sensory, electrical, magnetic, or cognitive means [70]. Stimulation of the nervous system triggers the transmission of neural signals, which can be recorded as EPs from various points along with the stimulated pathways [71]. EPs are typically characterized by their poststimulation latency in milliseconds (the time between the application of a stimulus and the occurrence of a peak in the EP waveform) and peak-to-valley amplitude (measured in millivolts or microvolts) in the waveform [70]. They are mainly categorized into motor-evoked potentials and sensory-evoked potentials. Sensory evoked potentials are further categorized into specific types, such as somatosensory evoked potentials (SSEPs), auditory evoked potentials (AEPs), and visual evoked potentials (VEPs).

Although evoked potential monitoring can assess the integrity of neural pathways, different results of interpretation often occur due to different warning criteria used for predicting detrimental outcomes. More strict criteria tend to increase the probability of false positives and thus decrease sensitivity, which interferes with the judgment of physicians and may lead to unnecessary treatments. In contrast, a too-low standard can cause failure to detect deteriorated conditions in patients, thus delaying the optimal time for treatment. There is no universally accepted standard for EPs due to the variability caused by surgical injury, ischemia, medications, and various physiological factors, such as hypothermia, hyperventilation, and hemodilution. Anesthetic drugs and sedatives are common pharmacological factors that can induce nonspecific changes in EPs [71].

Different monitoring modalities may have specific applications. Multimodal intraoperative neurophysiological monitoring ensures predictive and therapeutic effects on neurological functions. A summary of the recommendations for monitoring modalities is provided in Table **1**.

**Fig. (1):**
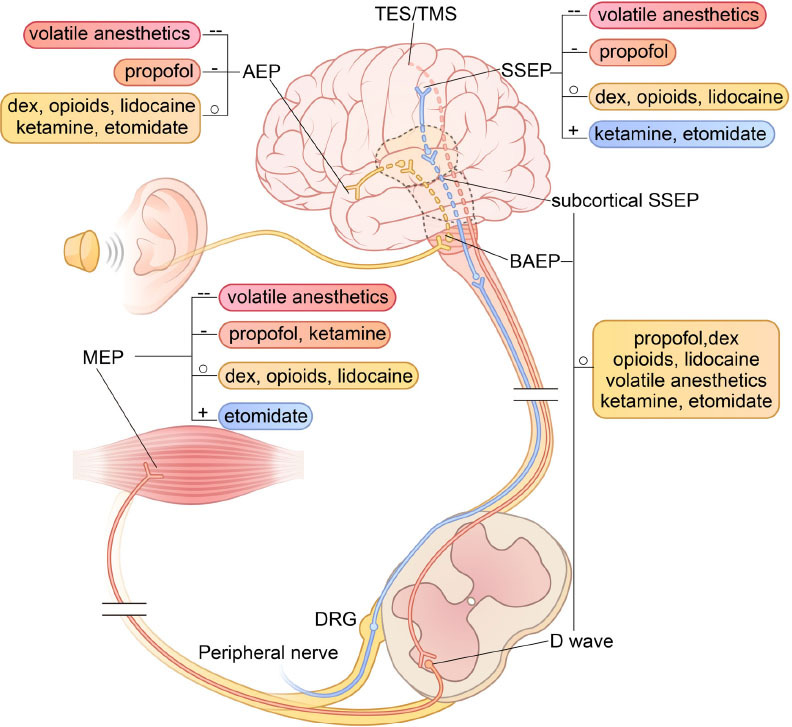
Effects of anesthetics and analgesics on evoked potentials.

In general, the effect of anesthetics on synaptic transmission is greater than that on nerve impulse conduction *per se* [72]. The recording modalities that contain more synapses are more sensitive to anesthetic drugs, and these effects are dose/concentration dependent. Moreover, intravenous anesthetics such as propofol have less of an impact on evoked potentials than volatile anesthetics (Fig. **1**).

Although anesthetic drugs affect neurophysiological monitoring intraoperatively, many other physiological or pathological factors can also affect neurophysiological monitoring. Reliably evoked potentials depend on the capacity of neurons and neuronal pathways to transmit and conduct electrical signals, which are correlated with optimal hemodynamic and respiratory parameters, volume status and hematocrit, electrolyte, and glycemic balance, and temperature control mechanisms of the entire body [73]. Maintaining homeostasis is, therefore, a prerequisite for recording reliable EP signals.

### 4.1 Motor Evoked Potentials

Motor Evoked Potentials (MEPs) are utilized to evaluate the motor component of the nervous system by capturing the response of the corresponding innervated muscle or nerve [74, 75]. This is achieved through techniques such as transcranial magnetic stimulation (tcm-MEP) or transcranial electrical stimulation (tce-MEP) of the motor cortex. MEPs allow for the monitoring of the integrity of downstream motor pathways that span from the internal capsule, brainstem, and spinal cord to the peripheral nerves and muscles. Common applications of MEPs include surgeries that involve aortic aneurysms, intramedullary spinal cord tumors, spinal deformities, posterior fossa tumors, intracranial aneurysms, and/or peri-rolandic brain surgeries [76]. MEPs can be recorded at the spinal cord level, termed D-waves, which indicate the direct activation of corticospinal axons, followed by a series of I-waves indicating the activation of synaptic circuits in the motor cortex [76]. Additionally, compound muscle action potentials (CMAPs) can be recorded within muscles using fine wire electrodes. Standard electrode montages for recording MEPs typically involve the Abductor Hallucis Brevis (AHB) and tibialis anterior (TA) for the lower extremities and the abductor pollicis brevis (APB) for the upper extremities [76, 77].

A reduction in the D-wave amplitude, unexplained by confounding factors, suggests a partial failure of corticospinal tract conduction above the recorded level. In spinal cord surgery, a 50% decrease in the D-wave amplitude is often considered a warning sign for intraoperative intervention [78]. Patients who experience D-wave loss during spinal cord surgery typically suffer permanent paraplegia [79]. Furthermore, a decrease of more than 50% in motor-evoked potential amplitude during tumor removal should be considered a serious warning sign during surgery [80]. There is no universal standard regarding the pathological significance of a reduction in muscle MEP magnitude.

### 4.2 Effects of Anesthetics on MEPs (Table 2)

#### 4.2.1 Propofol

Propofol produces a dose-dependent inhibition on MEP amplitude with limited effects on latency. MEP can also be monitored under appropriate doses of propofol, thus decreasing neurologic morbidity [3, 81]. Propofol may inhibit presynaptic neurotransmission by enhancing GABA_A_ receptor-mediated tonic inhibition in spinal ventral horn neurons [82, 83], thus suppressing the H-reflex (a monosynaptic stretch reflex) and F-wave (a late motor response following electrical stimulation of a nerve) amplitudes. However, this inhibition does not largely contribute to the inhibitory effect of propofol on tce-MEP amplitude due to preserved H-reflex and F-waves with reduced MEP amplitude when a stronger stimulus is utilized [84]. Instead, this suggests that propofol may inhibit supraspinal excitatory neurotransmission more strongly than it inhibits spinal motor neurotransmission, which involves less synaptic transmission.

#### 4.2.2 Dexmedetomidine

When dexmedetomidine, a selective α2 agonist, is used as an adjunct for general anesthesia, MEPs and SSEPs can be well maintained; therefore, dexmedetomidine can be safely used to monitor EPs [85-88]. This indicates that α2 agonism may leave motor pathways virtually unaffected at the mechanism level. However, a high bolus of dexmedetomidine might significantly attenuate the amplitude of tce-MEP [89-91]. This may be explained by the fact that dexmedetomidine can influence the muscle’s own reaction capacity directly or indirectly with increasing depth of sedation [92].

#### 4.2.3 Ketamine

Ketamine has been shown to have no significant influence on the amplitude of MEPs, suggesting that ketamine may be a suitable supplement to general anesthesia [93, 94]. The amplitude of the CMAP corresponds to the number of α-motor neurons activated, and it is plausible that ketamine leads to greater motor neuron firing in response to each transcranial magnetic stimulus, which results from the enhanced activity of cortical and/or spinal inter-neuronal transmission [95]. The effects of maintenance or even an increase in muscle tone by ketamine may contribute to the mild or no amplitude depression of tce-MEP [95]. However, a dose of ketamine ≥1 mg/kg may reduce the MEP amplitude by directly inhibiting excitatory synaptic neurotransmission. This highlights the importance for anesthesiologists to carefully consider the dosage and timing of ketamine administration [96]. In addition, ketamine can decrease conduction velocities by inhibiting sodium currents in guinea pigs and increasing the latency of spinal cord-evoked potentials in dogs [97, 98]. However, in humans, the peak latencies of MEPs are unchanged after ketamine administration [96], which contradicts findings in animals. In fact, increased MEP onset latency in humans may reflect a decrease in the number of activated motor units but not a reduction of conduction velocities [96]. However, further research is needed to confirm the mechanisms underlying the effect of ketamine on neural conduction velocity.

#### 4.2.4 Etomidate

Etomidate produces more favorable effects than propofol during MEP monitoring under comparable BIS levels [99, 100], as a lower stimulus intensity can induce an optimal waveform under etomidate. The effect of etomidate is thought to arise from its unique actions on the motor system at the spinal and supraspinal levels [101]. Generally, etomidate disinhibits subcortical structures, including the extrapyramidal system, brain stem, and/or spinal cord, thereby increasing the excitability of the motor system [99, 101, 102]. Etomidate may also increase cortical excitability by disrupting the balance between excitatory and inhibitory neuronal firing in the cortex. Additionally, increased motoneuronal excitability, monosynaptic reflex facilitation, enhanced electromyographic activity, minimal subcortical depression, and a lack of neuromuscular depression all contribute to the enhancing effect of low-dose etomidate on MEPs [102, 103]. Therefore, the relatively preserved function of the spinal cord in comparison to the cortical impacts renders etomidate superior to volatile anesthetics during MEP monitoring. At higher doses, etomidate reduces cortical excitability, and this effect may contribute to the diminished efficacy of descending electrical activity in generating the temporal summation required at the anterior horn cell to produce a CMAP response. However, the disappearance of the CMAP response in the presence of I-waves suggests that etomidate at high doses may depress spinal anterior horn cells *via* GABAergic mechanisms [100, 104].

#### 4.2.5 Neuromuscular Blocking Agents

Neuromuscular blocking agents (NMBAs) are commonly used in the perioperative period to assist with endotracheal intubation and to facilitate mechanical ventilation [105]. Currently, the widely used NMBAs mainly include succinylcholine, vecuronium, rocuronium, and/or cisatracurium. Each drug can paralyze muscle function by blocking the transmission of acetylcholine through neuromuscular junctions [105]. The onset of the action of succinylcholine, a depolarizing muscle relaxant, is rapid, and its duration is short, thus making it a suitable choice for rapid tracheal intubation.

One previous study revealed the TOF (Train of Four, measured by the number of detectable muscle contractions, with TOF(1) indicating deep blockade) can be used to characterize the level of muscle relaxation and to guide the use of NMBAs during surgery [106]. However, the deep neuromuscular block is incompatible with MEP monitoring. Neuromuscular blocking agents significantly decrease MEPs in a dose-dependent manner, with no significant changes in the latency of compound muscle action potentials (CMAPs) between patients with an anesthesia status of TOF(4) and those with an anesthesia status of TOF(2). Therefore, it is preferable to maintain a TOF value of not less than 2 when MEP monitoring is necessary [105].

Spontaneous neuromuscular recovery is often recommended before obtaining a baseline MEP after the administration of intubating doses of muscle relaxants or to avoid using NMBAs during MEP monitoring. Since the muscle response of MEPs is reduced by NMBAs, an approach to increase the MEP amplitude is required to obtain an optimal MEP recording. A study revealed that tetanic stimulation prior to MEP stimulation may potentiate MEP responses under NMBAs [107]. Sugammadex, a rapid NMBA reversal agent, reverses neuromuscular blocking by forming a very tight water-soluble complex with rocuronium in the plasma. This facilitates the release of rocuronium from cholinergic receptors, thus restoring the action of acetylcholine in the neuromuscular junction [108]. The administration of sugammadex provides rapid reversal of profound neuromuscular blockade by rocuronium and allows for MEP monitoring during spinal surgery [109, 110]. In total, partial neuromuscular blockade is a good option when there is a need to enable reliable MEP recording. Moreover, this approach can minimize patient movement to ensure surgical accuracy and stability.

#### 4.2.6 Volatile Anesthetics

Volatile anesthetics potently inhibit amplitudes of motor-evoked potentials and increase motor latency in a dose-dependent manner. Even low concentrations of sevoflurane have a significant suppressive effect on MEP amplitudes compared with baseline [111]. Isoflurane and desflurane produce modulatory effects on MEPs similar to those of sevoflurane due to their similar mechanisms of action. Evoked potentials, especially those involving multiple synaptic connections, are particularly susceptible to the influence of volatile anesthetics because of their potent ability to inhibit synaptic transmission. In addition, balanced anesthesia, which combines volatile and intravenous agents, might be associated with a significantly greater rate of false-positive MEP changes than TIVA (total intravenous anesthesia) during spinal surgery, which is probably related to the greater inhibition of MEPs by volatile anesthetics that decreases the amplitudes and increases the coefficient of variation [112].

Volatile anesthetics not only enhance the activity of GABA receptors but also suppress the activity of NMDA receptors, thus strongly inhibiting MEPs. The lower limbs appear to be more sensitive to volatile anesthetic-induced depression of MEPs than the upper limbs during MEP monitoring [113]. This difference might be related to the diversity in cortical spinal cord drive mechanisms between the upper and lower limbs, for example, D-wave refractory periods [114]. The longer refractory periods of D-waves in the lower limbs may render them comparatively less sensitive to external stimuli. Volatile anesthetics also cause muscle relaxation, which is consistent with the observation that the MEPs recorded in the spinal cord are diminished during volatile anesthesia [115]. Volatile anesthetics can inhibit neuronal excitability of the spinal anterior horn to a smaller extent than synaptic transmission of corticocortical projections, making D-waves comparatively insensitive to volatile anesthetics compared to I-waves.

#### 4.2.7 Local Anesthetics

The classification of local anesthetics is based on their structural differences and can be divided into two main categories: amino esters and amino amides. Local anesthetics exert neural inhibition mainly by blocking voltage-gated sodium channels. However, other ion channel targets of local anesthetics, such as K^+^ channels and Ca^2+^ channels, are gradually being identified [116-118]. Amino esters, such as procaine and tetracaine, are believed to exhibit lower systemic absorption due to their rapid clearance from plasma. In contrast, amino amides (*e.g*., lidocaine and bupivacaine) are metabolized by hepatic CYP (CYP450) enzymes in the liver [119]. The infusion of lidocaine can produce systemic analgesia and sedation, thus allowing for a reduction in the total dose of other intravenous anesthetics/sedatives [120]. One study emphasized that lidocaine infusion may not significantly affect MEPs due to its ability to reduce the need for propofol [121]. In contrast, epidurally administered ropivacaine lowered the amplitude of motor-evoked potentials and prolonged the onset latencies of both motor-evoked potentials and SSEPs in a concentration-dependent manner, which is probably related to the blockade of nerve conduction in the corticospinal tract and the inhibition of excitatory neurotransmission in the spinal ventral horn [122]. A high dose of ropivacaine used in epidural administration is not recommended if MEP monitoring is needed. Because high concentrations of ropivacaine may diffuse into the cerebrospinal fluid after epidural administration and then directly inhibit nerve conduction in the spinal cord, which suppresses MEPs [123]. Plane blocks and nerve blocks in regional anesthesia generally have a minimal impact on MEPs [124-126]. Blocks such as the Erector Spinae Plane Block (ESPB) primarily provide localized analgesia by inhibiting somatic sensory and motor nerve transmission. However, their direct influence on the spinal cord is typically limited. A pivotal study by Pan *et al*. demonstrated that the use of surgically-placed ESP catheters for local anesthetic delivery during posterior spine fusion surgeries did not result in significant interference with MEPs [125]. The mechanism of action for ESPB involves the spread of local anesthetic to the dorsal and ventral rami of the spinal nerves. While there is potential for the anesthetic to reach the epidural space, cadaveric studies indicate that this spread is inconsistent [127]. ESPB mainly functions as a sensory block, with the anesthetic spreading along sensory nerve roots to provide analgesia for somatic and visceral pain while largely sparing the deeper motor fibers. This sparing of motor pathways might minimize the risk of MEP disruption. However, a recent case report indicated that ESPB may cause a transient and significant deterioration of MEPs [128]. This could be attributed to the interaction between lower motor neurons, interneurons, and upper motor neurons at the spinal cord level. When ropivacaine is injected at the thoracic or lumbar levels, the anesthetic is more likely to diffuse widely, potentially affecting the synaptic transmission. This results in fewer action potentials, leading to a reduction in MEP amplitude, likely due to decreased synaptic activity and transmission efficiency [129].

In summary, most anesthetics potently inhibit polysynaptic transmission but less strongly suppress intrinsic neuronal excitability. This pharmacological characteristic makes the neural pathways that bypass poly-synapses (*e.g*., D-waves) less affected by anesthetics. Different anesthetics have different molecular targets and mechanisms of action, which leads to their distinct effects on motor-evoked potentials. Ketamine and etomidate may increase motoneuronal excitability by disrupting the balance between excitatory and inhibitory neuronal signaling, while propofol and other GABAergic agonists/enhancers strongly depress cortical and thalamic networks. The specific circuit involved in this depressive effect is complex, and how anesthetics influence MEPs at the molecular, cellular, and/or network levels still needs further investigation.

### 4.3 Somatosensory Evoked Potentials

The most widely used neurophysiological monitoring method in the clinical setting is somatosensory evoked potential (SSEP) monitoring [70]. Typically, a stimulus ranging from 0.2 to 2 ms that is applied to a peripheral sensorimotor mixed nerve elicits minimal muscle contraction and SSEPs. Common sites for stimulation include the median nerve at the wrist, the common peroneal nerve at the knee, and the posterior tibial nerve at the ankle. Neural signals then travel from the stimulated nerve through the dorsal root ganglion and/or dorsal column in the spinal cord, medial lemniscus, thalamus, and finally to the sensory cortex.

SSEPs consist of short-, mid-, and long-latency evoked potentials, depending on the placement of the recording electrodes. The subcortical (short-latency) component of SSEPs is typically recorded over the second cervical vertebra as a negative deflection (N-14), occurring ~14 ms after median nerve stimulation. The earliest cortical (mid-latency) component of the SSEP wave originates from the primary somatosensory cortex. It appears approximately 20 ms after median nerve stimulation (Table **3**) and ~40 ms after posterior tibial nerve stimulation [71]. SSEPs are significantly influenced by anesthetic drugs due to extensive synaptic connections within the cortex. Therefore, appropriate anesthesia regimens are necessary to obtain clinically useful recordings. Intraoperative monitoring of the subcortical SSEPs recorded over the second cervical vertebra can be particularly valuable due to preserved signals during deep anesthesia. Significant changes in SSEPs include a decrease in amplitude exceeding 50% and/or a delay in latency of more than 10% from baseline. After excluding confounding factors, both physiological and pathological changes should be considered when interpreting the results of SSEP recordings.

### 4.4 Effects of Anesthetics on SSEPs (Table 2)

#### 4.4.1 Propofol

Propofol dose-dependently decreases the SSEP amplitude and increases the latency during anesthesia. Propofol anesthesia with remifentanil is superior to volatile agents for cortical SSEPs but not for subcortical SSEPs [130-140]. Because desflurane/remifentanil anesthesia causes greater suppression of cortical SSEPs than cervical SSEPs under a comparable depth of anesthesia. Propofol can suppress the excitability of spinal sensorimotor neurons by inhibiting L-type calcium channel plateau potentials. In the brain, propofol enhances the inhibitory activity of GABA_A_ and decreases cortical synaptic transmission, thereby contributing to the depressive effect on SSEPs.

#### 4.4.2 Dexmedetomidine

Generally, dexmedetomidine has minimal or no effect on SSEPs and, therefore, can be safely used as an adjunct during EP monitoring [85-88]. Although dexmedetomidine can influence the later cortical peaks of somatosensory evoked potentials (SSEPs), reliable and reproducible potentials can be recorded under moderate-dose dexmedetomidine. At the mechanism level, dexmedetomidine primarily affects the locus coeruleus, which has no direct inhibitory effect on the cortex. Therefore, dexmedetomidine had no significant effect on cortical SSEPs.

#### 4.4.3 Ketamine

Interestingly, ketamine has been shown to increase the amplitude of cortical SSEPs, suggesting potential benefits for SSEP monitoring under low-dose ketamine anesthesia [93, 94]. However, the underlying mechanisms remain unclear. Speculatively, ketamine may preferentially inhibit NMDA receptors on GABAergic inhibitory interneurons, thereby disinhibiting downstream excitatory neurons.

#### 4.4.4 Etomidate

Although etomidate and propofol are both GABA_A_ receptor agonists/enhancers, etomidate has significantly different effects on evoked potentials than propofol. Speculatively, etomidate acts on various subtypes of GABA_A_ receptors, potentially leading to alterations in the balance of inhibitory and excitatory neurotransmission within the central nervous system, thereby resulting in distinct effects on SSEPs compared to those of propofol. A low dose of etomidate can increase the amplitude of SSEPs while exerting minimal influence on latency [141-147]. Another study indicated that the transient increase in the SSEP amplitude caused by etomidate is the initial response to oxygen deprivation.

Hyperpolarized cortical neurons are more sensitive to hypoxia than neurons in subcortical regions. Hence they amplify cortical waves [148].

#### 4.4.5 Opioids

Although a high dose of fentanyl may induce minor yet significant changes in SSEPs, SSEPs remain consistently and reliably monitorable during fentanyl analgesia [149-159]. It is important to note that continuous infusion of opioids at low doses may exert lesser effects on SSEPs than intermittent bolus injection [160]. In addition, SSEPs are not affected by intrathecal fentanyl, indicating that the changes in SSEPs associated with intravenous opioids are more likely due to their modulations at the supraspinal level [158].

#### 4.4.6 Neuromuscular Blocking Agents

Neuromuscular blocking agents do not directly influence sensory evoked potentials, suggesting that neuromuscular blockade may not contribute to changes in SEPs and that other physiologic and pharmacologic factors should be considered. Actually, SSEP recordings may be improved by reducing electromyography interference by neuromuscular blocking agents [161-182].

#### 4.4.7 Volatile Anesthetics

Compared with baseline, low concentrations of sevoflurane (~0.3 MAC) have no significant suppressive effect on either the latency or amplitude of SSEPs in the lower limbs [111]. However, the suppression of SSEPs by sevoflurane is enhanced with increasing concentrations of the end-tidal agent sevoflurane [129]. Multiple molecular targets may contribute to the greater inhibitory effects of volatile anesthetics on SSEPs at high concentrations. Moreover, preserved subcortical SSEPs suggest that volatile anesthetics have a more pronounced impact on polysynaptic pathways in the cortex than on the spinal cord conduction pathway. Balanced anesthesia with ~0.5 MAC of desflurane can be used in some patients during spine surgery utilizing SSEP monitoring. However, in certain patients, it may be advisable to avoid their use in favor of propofol–opioid TIVA [183].

#### 4.4.8 Local Anesthetics

Systemic administration of lidocaine does not affect the amplitude of subcortical SSEPs but may minimally prolong latency, while cortical SSEPs are profoundly influenced [184]. Intravenous infusion of lidocaine can help to reduce the total dose of other anesthetic agents. This anesthetic-sparing effect may account for the preserved subcortical SSEP amplitude [121]. The possible mechanism is that lidocaine enhances the monosynaptic reflex at the spinal level but preferentially suppresses multi-synaptic pathways in the cortex and/or supraspinal region. However, epidurally administered ropivacaine concentration-dependently inhibits SSEPs [122], probably by blocking large myelinated A-fibers and C-fibers. This effect leads to inhibition of the spinal dorsal horn and reduces postsynaptic depolarization mediated by NMDA and neurokinin receptors [158, 185]. Nerve blocks, such as brachial plexus and cervical sympathetic blocks, have been shown to affect SSEPs by altering the sensory pathways [186, 187]. SSEPs are critical for intraoperative monitoring of sensory nerve integrity, particularly during surgeries where real-time feedback on neural function is essential. The administration of local anesthetics can disrupt sensory signal transmission, leading to diminished SSEP amplitudes and delayed latencies. For instance, brachial plexus blocks have been associated with significant reductions in SSEP amplitude, potentially complicating the intraoperative monitoring of nerve function in upper extremity surgeries [186]. The effect of nerve blocks on SSEPs is largely contingent upon the anesthetic’s spread and concentration. Local anesthetics, such as bupivacaine and ropivacaine, exert their action by inhibiting voltage-gated sodium channels, which are pivotal for the propagation of action potentials in sensory and motor neurons. By impeding these channels, anesthetics diminish the excitability of peripheral sensory nerves, thereby reducing the conduction of sensory signals to the central nervous system. This interference manifests as reduced SSEP amplitudes and prolonged latencies during neurophysiological monitoring [186, 188]. Despite the potential for SSEP signal attenuation, nerve blocks remain invaluable for providing intraoperative analgesia. Further investigations into optimizing the dosing and delivery of anesthetics may offer a balance between effective pain management and the preservation of reliable SSEP monitoring.

In summary, anesthetics can block sensory signal transmission from peripheral nerves at various locations. Monitoring of SSEPs at different sites allows the identification of major blocking sites. In general, most systemically administered anesthetics profoundly inhibit cortical components of SSEPs. However, agents such as etomidate and ketamine can be beneficial for SSEP monitoring. Further work is needed to address the limitations of current technologies and methodologies. For example, better electrode design and/or signal processing algorithms may improve the variability of SSEPs within patients and, more importantly, the sensitivity and specificity of neurological outcomes.

### 4.5 Auditory Evoked Potentials

Auditory Evoked Potentials (AEPs) are utilized to evaluate the integrity of auditory conduction pathways following sound stimulation during surgery. These evoked potentials are categorized into transient and steady-state subtypes. Transient AEPs reveal evoked potentials with varying latencies along the auditory pathway. The brainstem components of transient short-latency AEPs (<10 ms) are termed brainstem auditory evoked potentials (BAEPs) or auditory brainstem response (ABR), which are used to assess the functional status of the brainstem [71]. Transient BAEPs are typically recorded using a recording electrode positioned near the mastoid or in the ear. The reference electrode is placed at the top of the head, while the ground electrode is positioned at the forehead [70]. BAEPs are particularly valuable for evaluating the structural integrity of the brainstem during surgical procedures involving the posterior cranial fossa, such as the resection of acoustic neuromas, cerebellopontine tumors, and microvascular decompression of the trigeminal and facial nerves [189, 190].

Long-latency (>10 ms) auditory evoked potentials (AEPs), comprising early cortical waves (10-100 ms) or middle latency waves and late cortical waves (>100 ms), are used to assess cortical function. Early cortical waves, including No, Po, Na, Pa, and Nb (P for positive and N for negative), originate from the medial geniculate and primary auditory cortex (Table **3**). Late cortical waves, such as P1, N1, P2, N2, and P3, are generated from the frontal cortex and associated areas [191, 192].

The frequency of the sound stimulus influences the amplitude of the evoked response. When the stimulus frequency reaches or exceeds 40 Hz, it produces steady-state AEPs known as 40 Hz or gamma rhythms, which are implicated in increased cognitive function [193]. The 40-Hz steady-state response diminishes under anesthesia but reappears upon recovery [193-195]. A reduction in amplitude of at least 50% and/or an increase in latency of at least 1 ms are commonly considered indicative of intraoperative neural damage and postoperative hearing loss risk [190].

### 4.6 Effects of Anesthetics on AEPs

#### 4.6.1 Intravenous General Anesthetics

Propofol can reversibly increase the latency of the middle-latency auditory evoked potential (MLAEP) [142]. Therefore, MLAEP recording seems to be a promising method for monitoring the level of anesthesia as defined by spontaneous movement during anesthesia [143]. The brainstem response remains intact due to relatively less synaptic transmission compared with that of MLAEPs. Despite its effects on MEP amplitude, auditory evoked responses remain well maintained during ketamine administration, serving as practical indicators for monitoring the integrity of the ascending auditory pathway [196-198]. Ketamine does not induce a general suppression of the perception and processing of sensory stimuli, which contributes to the preservation of MLAEP. Nonetheless, unaffected MLAEP under ketamine anesthesia indicates a greater incidence of intraoperative awareness [153].

The effect of etomidate on auditory evoked responses is also minimal, which indicates that the brainstem response is unsuitable as a universal method for measuring the depth of anesthesia [149] but can be used as a reliable indication of brainstem auditory function under etomidate anesthesia. Additionally, the changes in MLAEP during propofol anesthesia appear to be unaffected by the presence of alfentanil, rendering MLAEP monitoring feasible even in the presence of opioids [161, 199]. Neither intravenous nor epidural lidocaine has a significant effect on AEP, which suggests that lidocaine can be used for AEP monitoring to ensure successful neurophysiological function measurement [173, 174].

MLAEPs are only slightly altered in amplitude and latency during the induction of general anesthesia with benzodiazepines, and primary cortical processing of auditory stimuli seems to be preserved under benzodiazepines. Although benzodiazepines show amnesic effects during surgery, the preservation of AEP increases the probability of intraoperative awareness [165, 200]. The effect of diazepam on evoked potentials remains contentious, and a few studies have demonstrated significant changes in evoked potentials following the administration of diazepam [167, 201]. Further investigation is warranted to elucidate the effects of diazepam on evoked potentials and its utility in postoperative physiological function judgments.

#### 4.6.2 Volatile Anesthetics

MLAEPs exhibit a significant decrease in amplitude and/or an increase in latency under sevoflurane anesthesia in a dose-dependent manner [131]. At concentrations of approximately 1.5-2.0 vol% sevoflurane (0.75-1.0 MAC), MLAEPs are severely attenuated or even abolished, but the initial transduction of auditory stimuli remains intact [202]. Because MLAEPs are generated in the primary auditory cortex of the temporal lobe, we can infer that auditory information can be transmitted to the brainstem or midbrain but not to the auditory cortex under sevoflurane anesthesia [202, 203]. A similar effect on MLAEPs has been observed among other volatile anesthetics, such as isoflurane, enflurane, and halothane [203-206]. Therefore, the suppression of MLAEPs may serve as a sign of the loss of auditory perception and consciousness to avoid intraoperative awareness. One study reported that after halothane withdrawal, the early cortical components recovered partially, while the brainstem components remained inhibited [204]. This phenomenon, unexplained by brainstem to cortical transmission, might be caused by the differential sensitivity of the various brain regions to halothane.

### 4.7 Visual Evoked Potentials

Visual Evoked Potentials (VEPs) are recorded from scalp electrodes positioned over the occipital and parietal areas following stimulation of the retina [207]. Intraoperative monitoring of VEPs seems to have limited application compared to other evoked potentials. The characteristic waves of flash VEP include a negative wave with a latency of 75 ms (N75) and a positive wave with a latency of 100 ms (P100), with P100 originating in the occipital cortex and serving as a crucial marker for intraoperative monitoring [70]. Changes in the amplitude of the VEP are considered more reliable indicators of neural damage than changes in latency in the operating room, as latency tends to be more influenced by anesthesia. Intraoperative VEPs have demonstrated sensitivity in detecting vascular damage during aneurysm clipping and mechanical manipulation of the anterior visual pathway at an early reversible stage [208, 209].

In general, all volatile anesthetics markedly prolong the VEP latency and decrease its amplitude in a dose-dependent manner [209]. Therefore, volatile anesthetics are not preferred when monitoring VEPs. Compared to volatile anesthetics, the use of TIVA resulted in greater VEP amplitudes and shorter latencies [210]. Anecdotally, a recent study demonstrated that 0.5 MAC sevoflurane combined with propofol and remifentanil produced a non-inferior effect on the P100-N145 amplitude compared with that under propofol-based total intravenous anesthesia [211]. Sometimes, it is also difficult to record VEPs under total intravenous anesthesia [212], probably because VEPs involve more synaptic transmissions; thus, they become more unstable and more prone to alteration by anesthetic agents than other evoked potentials.

An animal study revealed that the change of the VEP under sevoflurane shows an increase in the latency of wave N1 and a slight rise in latency P1 with the increase in the depth of the anesthesia. However, the duration between the N2 wave and P1 wave decreases, which may indicate increased intracortical communication of sevoflurane anesthesia that is not found in chloral hydrate [133]. Thus, evoked potential monitoring may also have a potential role in probing the mechanism of action of anesthetic drugs.

### 4.8 Electromyography (EMG)

Intraoperative electromyography (EMG) monitoring serves various purposes, mainly including monitoring the muscles innervated by cranial and peripheral nerves, assessing conduction integrity, and localizing target nerves. Typically, electromyography signals are recorded using paired intramuscular needles or wire electrodes, which are inserted after the patient is anesthetized and before surgery [213].

Spontaneous EMG detects mechanical and/or metabolic nerve irritation and can be obtained from innervated muscles without electrical stimulation by monitoring the nerve roots at risk [3]. The recognition of abnormal high-frequency EMG activity enables surgeons to identify the effects of blunt mechanical injury on nerve roots and peripheral nerves, thereby preventing severe irreversible damage. Stimulus-evoked EMGs, such as compound muscle action potentials (CMAPs), aid in identifying uninjured nerves and preventing medically induced injury. Nerve conduction studies (NCSs) offer valuable quantitative and qualitative insights into neuromuscular function. They are particularly useful in evaluating patients with suspected neuromuscular disorders and complement other elements of electrodiagnostic evaluation [214].

The effects of anesthetics on EMGs were mainly discussed in the MEPs section earlier in this review. Briefly, total intravenous anesthesia (TIVA) is often utilized when electrophysiological monitoring is used during surgery. This is because TIVA has a more moderate effect on neurophysiological parameters than volatile anesthetic while maintaining undisturbed movement intraoperatively. However, a meta-analysis reported that TCI (target-controlled in fusion) of a high concentration of remifentanil and a relatively low concentration of propofol without volatile anesthetics resulted in a low incidence of spontaneous movement, which is probably due to the activation of glycine receptors in the opioid formulas [215].

Compared with TIVA, combined intravenous and volatile anesthesia with remifentanil prolonged the time to the first positive EMG signal during IONM in patients undergoing thyroidectomy, suggesting that TIVA may be more favorable for the early detection of IONM signals [216].

### 4.9 DBS-related Electrophysiologic Monitoring

#### 4.9.1 Deep Brain Stimulation

Deep Brain Stimulation (DBS) surgery is extensively performed to treat various movement disorders, including Parkinson's disease, tremor, dystonia, epilepsy, Tourette's syndrome, and obsessive-compulsive disorder [217]. It offers a safer long-term treatment option than neuroablation due to its controllability and adjustable stimulation parameters [217, 218]. Surgeons utilize a combination of image-guided stereotactic techniques and intraoperative neurophysiology to accurately and safely locate surgical targets for DBSs. Five common electrophysiological techniques are employed for physiologic localization during DBS surgeries: impedance measurements, macro electrode recordings and stimulation, semi-microelectrode recording (semi-MER), microelectrode recording (MER), and local field potentials (LFPs) [219].

MER, the standard technique used in most centers for recording during DBS surgery, provides detailed information about the neural elements encountered during movement disorder surgery [220-224]. Microelectrodes used in neurosurgery typically have diameters ranging from 1 to 40 μm. These microelectrodes also exhibit impedances of approximately 1 MΩ. Their use provides valuable insights into individual neuronal activity, thereby enhancing stereotactic localization during procedures. LFP, comprising extracellular potentials from neurons surrounding the electrode, is utilized for target confirmation [225, 226] and optimization of DBS parameter programming postoperatively. Oscillatory activity within specific nuclei correlates with unique disease states; for example, peaks in the beta band (13-30 Hz) have been observed in Parkinsonian patients but not in those treated with L-DOPA or individuals without Parkinson's disease [227].

DBS disrupts abnormal information flow by decoupling input and output signals within the targeted nucleus [228]. The stimulating electrode may directly activate local axons and reach axon terminals orthodromically and antidromically, further affecting synaptic transmission at the axon terminals. However, the net effects of DBS at the circuit level depend on the neural composition of the stimulated nucleus. One prominent area of focus currently revolves around the impact of DBS on astrocytes, considering the intimate relationship between astrocytes and neurons [228-230].

The administration of propofol may be a confounding factor for the localization of the targeted MER area in DBSs due to dose-dependent suppression of spontaneous and evoked potentials [231]. Moreover, as with propofol, low concentrations of sevoflurane, desflurane, and isoflurane all appear to alter neuronal activity under DBS [232]. However, low-dose dexmedetomidine may exert minimal effects on MER [233]. In general, GABAergic anesthetic agents have been shown to influence both background activity and neuronal spike activity, while non-GABAergic drugs (dexmedetomidine, ketamine) and short-acting opioids (fentanyl, remifentanil) have negligible effects on MER [234]. The lack of high-quality studies emphasizes the need for prospective randomized controlled trials to clarify how anesthetic/analgesic drugs affect neurophysiological signal recordings during DBS surgeries.

#### 4.9.2 Functional Area Monitoring in Awake Brain Surgery

Awake brain surgery has emerged as a pivotal technique in modern neurosurgery, primarily due to its significant advantages in neurological and cognitive preservation [235, 236]. By keeping patients awake during surgery, neurosurgeons are able to perform intraoperative mapping of brain functions *via* electrical stimulation. This technique not only improves the accuracy of tumor resection but also protects critical brain areas. Furthermore, it offers significant insights into cognitive neuroscience by enabling direct observation of how surgical interventions impact cognitive and neurological function [237].

In addition to the previously discussed neurophysiological monitoring techniques, awake brain surgery also incorporates direct electrical stimulation (DES). Intraoperative mapping of language and sensorimotor functions using DES enables precise identification of functionally critical neuronal structures, making it a standard technique for resecting cerebral tumors that affect eloquent cortical areas and subcortical pathways [238]. The influence of anesthetic agents on cognitive testing during awake surgery is a key consideration in neurophysiological monitoring. Anesthetic regimens, particularly those involving propofol and opioids, are commonly utilized due to their ability to maintain patient responsiveness while minimizing discomfort [236]. However, these agents can affect cognitive assessments by altering the patient’s alertness and response times. Studies highlight the need to select cognitive tests that are appropriate for the patient’s condition and the surgical context, with digitalized tests providing precise outcome measures that can be particularly advantageous intraoperatively [236].

The impact of anesthetic agents on language function mapping during awake craniotomy is a critical area of research, as it directly affects surgical outcomes and patient safety. A careful balance is required when administering anesthetics to ensure patient comfort while allowing for accurate neurophysiological monitoring. Saito *et al*. emphasize the role of intraoperative cortico-cortical evoked potentials (CCEPs) in evaluating language function, noting that anesthetics can modify the responsiveness of cortical areas involved in language processing [239]. Several studies have reported reductions in CCEP amplitude under general anesthesia compared to the awake state [240, 241]. While the effects of propofol on MEPs have been previously discussed, the mechanisms by which anesthesia influences CCEPs and their generation remain to be fully elucidated.

Moreover, the combined use of DES and transcranial cortical stimulation (TCS) allows for the evaluation of both voluntary and involuntary movements, providing critical insights into motor function [238]. Surbeck and colleagues highlight that the combination of propofol and short-acting opioids remains the preferred regimen for awake craniotomies, offering a balance between sedation and rapid recovery [237]. This combination facilitates effective motor mapping by maintaining patient responsiveness while minimizing discomfort. The introduction of dexmedetomidine offers additional advantages, such as sedation and analgesia, which may enhance the mapping process by reducing patient stress and movement [237]. Continued investigation into the effects of anesthetic agents on intraoperative neurophysiological monitoring, as well as the underlying mechanisms, is vital. Furthermore, tailored anesthetic protocols that accommodate the specific requirements of both the surgical team and the patient are critical. Ongoing research and the refinement of these protocols are essential for enhancing the efficacy of neurophysiological monitoring during awake surgeries, thereby optimizing patient care and improving surgical outcomes.

### 4.10 H-reflex

The H-reflex is perhaps one of the simplest reflexes that can be recorded in human subjects by stimulating the peripheral nerve, and it has become a standard tool in sensorimotor integration assessment studies [242]. The H-reflex is a monosynaptic projection of group Ia (muscle sensory fibers) afferents onto homonymous motoneurons. Electrical stimulation of a mixed peripheral nerve above the motor threshold (MT) produces two responses in the homonymous muscle: an M-wave (a short-latency direct motor response due to stimulation of motor axons) and an H-reflex [242]. The M-wave amplitude is an important point of reference. It should be monitored so that its size is similar under all tested conditions, which will ensure that the change in the pattern is due to the mechanisms that act to inhibit or potentiate the H-reflex. When Ia afferents are stimulated, Ib inhibitory afferents are also stimulated unwantedly. This would confound the amplitude of the reflex response, notably when Ia excitation is not brief (in soleus motoneurons and the quadriceps femoris) [243, 244]. The H-reflex amplitude serves as a more sensitive measure of neuronal interactions than simple electromyography. Latency is measured from the start of the 1-ms stimulus to the take-off of the H wave from baseline rather than to the onset of negativity. Reflex latency varies with limb length (or height) and age [245-247]. Normally, the threshold of the reflex response relative to the M-wave is used as an indication because the amplitude of the reflex response is inconsistent between subjects.

The H-reflex has been used as a tool to investigate neuronal pathways and spinal inhibitory control systems closely linked to the neural regulation of movement in both healthy individuals and those with neurological disorders. [242]. For example, clinical conditions characterized by a reduced ankle reflex, such as polyneuropathy, sciatic neuropathy, or sciatic radiculopathy, typically exhibit a diminished or absent H reflex [248].

Previous studies have revealed that propofol can reduce the MEP amplitude while preserving the H-reflex and F-wave (following M-wave) amplitudes [84, 249]. This finding suggests preserved spinal motoneuron circuit excitability under propofol anesthesia. Transcranial electrical stimulation transmits signals *via* the monosynaptic and polysynaptic pathways, while the H-reflex and F-waves involve monosynaptic and no synaptic transmission, respectively. Thus, stronger inhibition of polysynaptic pathways by anesthetic drugs might explain the preserved H-reflex and F-wave. Notably, propofol can depress the H-reflex by inhibiting motor neurons [250]. The difference between those studies might be due to the stimulation intensity. A lower stimulating intensity might recruit smaller motoneurons that are more susceptible to propofol with a lower stimulating threshold. Therefore, when the stimulating intensity becomes stronger, the inhibitory effect of propofol on the H-reflex is reduced as a result of the recruitment of larger motoneurons that are relatively resistant to propofol [251]. Considering the specific neural transduction of the H-reflex, further research is needed to clarify the effects and underlying mechanisms of various anesthetics and/or analgesics.

## 5 OTHER EVOKED RESPONSES AND THE IMPLICATIONS FOR MECHANISMS OF ANESTHESIA

A common approach for understanding brain networks and their correlation with brain state is to examine responses to stimulation, which can be used to explain the mechanisms of anesthesia and to identify the neural correlates of consciousness [252-255]. For example, deep cortical stimulation may elicit a triphasic spiking pattern, a brief excitation followed by a period of silence and then a rebound excitation in local cortical and thalamic neurons only during the awake state. This may reflect a cortical-thalamic-cortical (CTC) interaction, which is reduced during isoflurane anesthesia [256].

Generally, higher-level, longer-latency evoked responses are more susceptible to anesthetics than short-latency evoked responses. This difference is reflected in the thalamocortical circuits being affected at low drug levels, while subcortical responses are reduced only at high drug levels. Despite the deepest levels of anesthesia when brain activity enters a state of burst suppression, sensory stimuli can still elicit bursts of whole-brain activity [257]. This phenomenon might be mediated by increased extracellular Ca^2+^ concentrations, which may transiently enhance cortical synaptic processes and appear to drive nonspecific cortical activity rather than effective sensory processing.

In previous studies on emergence from anesthesia, auditory stimuli were shown to evoke large potentials that are absent during induction [258], which is reminiscent of neuromodulatory arousal systems affecting only the emergence period [259], suggesting that neuromodulatory activity in mediating cortical and thalamic circuits might account for the unique evoked responses during emergence. Layer 6b is the only cortical layer responsive to orexin, a vital neuromodulator [260]. Photostimulation of layer 6b neurons suppresses oscillations most strongly in the delta and theta ranges in sleep-deprived mice while ceasing photostimulus immediately restores the slow oscillations [261]. Direct excitation of the higher-order thalamocortical system and recruitment of local excitatory-inhibitory loops in cortical layer 6 may contribute to the disruption of slow oscillations and enhancement of fast oscillations [262]. However, unidirectional excitation of the CTC loop by activation of layer 6b is not sufficient to counteract the temporary continuation inhibitory circuits, which could explain why the mice returned to baseline oscillation levels immediately after stopping photostimulation.

The different spatial and temporal characteristics of evoked responses depend on the state of consciousness. A possible future direction for understanding the mechanisms of anesthesia is to evaluate responses to internal or external stimulation during wakefulness, sleep, and various drug-induced losses of consciousness.

## 6 CLINICAL SIGNIFICANCE OF NEUROPHYSIOLOGICAL CHANGES

All IONM signal changes, including transient changes, should result in an evaluation of the patient's blood pressure, anesthesia regimen, and/or surgical procedure. Once these issues are addressed and scrutinized, preserved IOM signals are suggestive of preserved neurologic outcomes [262]. Regardless of the IONM modality used, sudden changes in electrophysiological signals might reflect impending neural damage and should always prompt immediate intervention by surgeons and anesthesiologists [263].

If the intraoperative neurophysiological monitoring warning criteria are met [264], even if no common standards are used, quickly and accurately identifying and correcting the potential cause is critically important. First, the reproducibility of the warning signal must be checked to prevent interference or equipment malfunction. The whole surgical team should be alerted to this problem and pause any further surgical manipulation if necessary. The reasons for the occurrence of signal changes can be categorized into the following three groups: physiological anesthetic factors, technical monitoring factors, and surgical factors.

Careful anesthesia management is required to reduce the incidence of false positive warnings, as anesthetic drugs can directly influence the quality of the signals. If the SSEPs or MEPs are compromised under balanced anesthesia of low-concentration volatile anesthetic and titrated intravenous anesthetics, an assessment of the depth of anesthesia may be performed due to the strong inhibition of synergistic interactions between anesthetics. Then, the volatile anesthetic can be either decreased or discontinued, and a TIVA strategy is substituted. Typically, increasing the dose of the opiate and reducing the dose of the other drugs known to depress EPs might help reemerge the signals [73]. If the neurophysiological signals are still not restored, physiological and other nonsurgical factors that might confound the signals should be taken into consideration, such as oxygenation, body temperature, and blood perfusion of the CNS, which can be influenced by ventilation and blood pressure. Hemodynamic management usually focuses on maintaining the mean arterial blood pressure within the cerebral autoregulation range (60-140 mmHg). Body core temperature predominantly affects MEP latency and should be maintained between 35°C and 37°C [265].

The monitoring technical factors mainly include incorrect electrode location, electrical noise from new equipment, machine malfunction, and test paradigm changes. Surgical factors, including blunt or surgical trauma, mechanical effects, vascular occlusion, and tissue resection, should also be considered. An acute signal alteration caudal to the level of surgical manipulation, with the cranial signals unaffected, strongly suggests a surgical cause of spinal cord injury, while a subacute global signal change suggests a systemic or anesthetic problem [266].

In the search for potential causes, anesthesiologists can help minimize potential neurological damage by optimizing vital signs (such as elevated blood pressure, if appropriate). Once the cause has been identified, it should be corrected as soon as possible. Further treatment may depend on follow-up monitoring and clinical conditions.

## CONCLUSION AND PROSPECTS

Intraoperative neurophysiological monitoring (IONM) serves as an objective and precise modality for assessing the neural function of patients with compromised conditions under general anesthesia, thus offering effective safeguards for patients at heightened risk of neurological injury. However, the administration of anesthetic agents during surgery can exert variable impacts on IONM parameters, potentially disrupting the accuracy of intraoperative neural monitoring. This review comprehensively describes the influence and underlying mechanisms of commonly utilized anesthetic agents on IONM parameters within clinical settings. Such elucidation not only aids in the judicious selection of anesthesia protocols to optimize monitoring outcomes but also informs surgical decision-making processes.

In the realm of diverse surgical interventions, the meticulous selection of an optimal anesthesia regimen is of paramount significance. However, a standardized warning criteria protocol for IONM is currently lacking. Additional investigations are warranted to elucidate the most suitable anesthesia modalities and monitoring approaches tailored to specific surgical contexts, with a view to augmenting both specificity and sensitivity. Future research endeavors may also focus on refining monitoring technologies and signal-processing algorithms. Such endeavors hold promise in facilitating expeditious feedback through streamlined monitoring modalities to manage intraoperative exigencies effectively. Various anesthetic drugs have distinct impacts on neurophysiological monitoring, potentially revealing diverse mechanisms of action. Moreover, leveraging neurophysiological monitoring allows for the exploration of the molecular mechanisms underlying different anesthetic drugs and their differential effects on sensory and motor circuits.

## Figures and Tables

**Table 1:** Recommendation of monitoring modalities for different surgeries.

**SSEP/MEP**	**BAEP**	**VEP**	**EEG**
Spine and spinal cord surgery	Acoustic neuroma resection	Optic nerve surgery	Carotid endarterectomy
Brain and brain stem surgeries	Vestibular nerve section	Orbital surgery	Cerebral aneurysm clipping
Cerebrovascular surgery	Vascular loop decompression	Pituitary gland surgery	Monitoring depth of anesthesia
Pelvic fracture surgery	Vestibular schwannomas	-	Epilepsy surgery
Thoracoabdominal aortic aneurysm repair	Facial nerve decompression	-	-
Repair of coarctation of the aorta	Brainstem tumor resection	-	-
Thyroid surgery	Auditory brainstem implant	-	-
Peripheral nerve repair	Posterior fossa procedures	-	-
Carotid endarterectomy	Assess auditory pathways within the brainstem	-	-
Brachial plexus and lumbosacral plexus surgery	Functional localization of the cortex with direct cortical stimulation	-	-
-	Assess ischemia at the cochlea and eighth nerve	-	-

**Note**: The data are summarized from [74]. This work is permitted for use under the terms of the Creative Commons Attribution-NonCommercial-NoDerivatives 4.0 International (CC BY-NC-ND 4.0). **Abbreviations**: SSEP: somatosensory evoked potential; MEP: motor evoked potential; BAEP: brainstem auditory evoked potential; VEP: visual evoked potential; EEG: electroencephalography.

**Table 2:** Effects of anesthetics on evoked potentials.

**Drugs**	**SSEPs**		**AEPs**		**VEPs**		**MEPs**	
	**Latency**	**Amplitude**	**Latency**	**Amplitude**	**Latency**	**Amplitude**	**Latency**	**Amplitude**
Sevoflurane	+ [129, 130]	- [129, 130]	EC: + [131]	EC:- [132]	+ [133]	- [130]	+ [111]	- [111]
Isoflurane	+ [134]	- [134]	EC: + [135, 136]	EC: - [136]	+ [137]	- [137]	+ [138, 139]	- [138, 139]
Desflurane	+ [140]	- [140]	EC: + [141]	EC:- [141]	-	-	+ [111]	- [111]
Propofol	+ [134]	- [134]	EC: + [142, 143] SC:NA [144]	EC:- [142, 143] SC:NA [144]	NA [145]	- [130]	NA [146]	- [84]
Etomidate	+ [147, 148]	+ [147, 148]	NA [149]	NA [149]	+ [150]	NA [150]	- [99]/ NA [151]	+ [99]/- [151]
Ketamine	NA [96]	NA [96]/+ [152]	NA [153, 154]	NA [153, 154]	NA [155]	- [155]	NA [95, 96]	- [96]
Dex	NA [85, 87-89, 156]	NA [85, 87-89, 156]	-	-	NA [85]	NA [85]	NA [85, 88]	NA [85, 88]
Opioid	NA [157, 158]/+ [148, 159, 160]	NA [157, 158]/- [148, 159, 160]	NA [157] LC: + [161]	NA [157] LC: - [161]	NA [157]/+ [162]	NA [157]/- [162]	NA [163]	NA [163]
Diazepine	NA [157, 164]	NA [157]/- [164]	NA [157, 165]	NA [157, 165]	NA [157]	NA [157]/- [166]	NA [167]	NA [167]
Midazolam	+ [168, 169]	- [168, 169]	NA [165, 170]	NA [165, 170]	+ [171]	-	NA [151]	- [151]
Lidocaine	NA [172]	NA [172]/AB [158, 163]	NA [173, 174]	NA [173, 174]	-	-	NA [172, 175]	NA [172, 175]/AB [163]

**Note**: “+”: enhance; “-”: inhibit; “NA”: no effect or minimal; “AB”: abolished; “EC”: early cortical; “SC”: subcortical; “LC”: late cortical; SSEPs: somatosensory evoked potentials; MEPs: motor evoked potentials; AEPs: brainstem auditory evoked potentials; VEPs: visual evoked potentials; Dex: dexmedetomidine.

**Table 3:** Anatomic representation of different waveforms in SSEPs (median nerve stimulation) and BAEPs.

**BAEP**	**Wave Name**	**Anatomic Representation**	**SSEP**	**Wave Name**	**Anatomic Representation**
Brainstem response	I	Acoustic nerve	Subcortical response	N9	Brachial plexus
	II	Cochlear nucleus		N11	Posterior columns or spinal roots
	III	Superior olivary complex		N13/P13	Dorsal column nucleus cuneatus
	IV	Lateral lemniscus		P15	Medial lemniscus
	V	Inferior colliculus	Early cortical response	N19	Thalamus or initial cortex
Early cortical response	VI	Medial geniculate and primary auditory cortex		P22	Primary cortex
	N0		-	-	-
	P0		-	-	-
	Na		-	-	-
	Pa		-	-	-
	Nb		-	-	-
Late cortical response	P1	Frontal cortex and association areas	-	-	-
	N1		-	-	-
	P2		-	-	-
	N2		-	-	-

**Note**: P15, N19, and P22 are recorded at the contralateral mastoid process. The data are summarized from [70, 196]. SSEP: somatosensory evoked potential; BAEP: brainstem auditory evoked potential; P for positive, N for negative.

## LIST OF ABBREVIATIONS

AHB: Abductor Hallucis Brevis
APB: Abductor Pollicis Brevis
ABR: Auditory Brainstem Response
AEPs: Auditory Evoked Potentials
BIS: Bispectral Index
BAEP: Brainstem Auditory Evoked Potential
CNS: Central Nervous System
CBF: Cerebral Blood Flow
CMAPs: Compound Muscle Action Potentials
CTC: Cortical-thalamic-cortical
DBS: Deep Brain Stimulation
DRG: Dorsal Root Ganglion
EEG: Electroencephalography
EMG: Electromyography
EPs: Evoked Potentials
GABA: Gamma-aminobutyric Acid
GIRKs: G-protein-gated Inward Rectifying Potassium Channels
HCN: Hyperpolarization-activated Cyclic Nucleotide-gated Channels
IONM: Intraoperative Neurophysiological Monitoring
LFPs: Local Field Potentials
LC: Locus Coeruleus
MER: Microelectrode Recording
MLAEP: Middle-latency Auditory Evoked Potential
MAC: Minimum Alveolar Concentration
MEPs: Motor Evoked Potentials
NCSs: Nerve Conduction Studies
NMBAs: Neuromuscular Blocking Agents
NMDA: N-Methyl-D-Aspartate
POA: Preoptic Area of the Hypothalamus
semi-MER: Semimicroelectrode Recording
SEPs: Sensory Evoked Potentials
SSEPs: Somatosensory Evoked Potentials
sEMG: Spontaneous Electromyography
TCI: Target-controlled in Fusion
TRN: Thalamic Reticular Nucleus
TA: Tibialis Anterior
TIVA: Total Intravenous Anesthesia
TOF: Train of Four
tce-MEP: Transcranial Electrical Stimulation Motor Evoked Potential
tcm-MEP: Transcranial Magnetic Stimulation Motor Evoked Potential
tEMG: Triggered Electromyography
VEPs: Visual Evoked Potentials
